# Supplementary material for: A novel assay for improved detection of sputum periostin in patients with asthma
Source: PLoS One. 2023 Feb 10;18(2):e0281356. doi: 10.1371/journal.pone.0281356 (PMC9916630; doi:10.1371/journal.pone.0281356)
Supplement: S1 Table — (DOCX) [file pone.0281356.s002.docx]

**S1 Table. Assay validation for Assay A and Assay B.**

|  | Assay A | | Assay B | |
| --- | --- | --- | --- | --- |
| Limit of blank (LOB) | 2.1 pg/ml  3.8 pg/ml  4.0 pg/ml  95-102%  r=0.999 | | 3.3 pg/ml  5.1 pg/ml  6.0 pg/ml  95-101%  r=0.999 | |
| Limit of detection (LOD) |  |  |  |  |
| Limit of quantification (LOQ) |  |  |  |  |
| Recovery rate |  |  |  |  |
| Linearity |  |  |  |  |
|  | Mean (SD), ng/ml | CV, % | Mean (SD), ng/ml | CV, % |
| Intra-assay  (n=20) | 1.22 (0.11)  5.31 (0.17)  46.8 (1.94) | 8.7  3.2  4.1 | 1.28 (0.11)  5.45 (0.22)  51.8 (1.79) | 8.8%  4.0%  3.5% |
| Inter-assay  (n=10) | 1.21 (0.03)  5.30 (0.93)  48.3 (1.9) | 2.5  3.6  1.9 | 1.28 (0.05)  5.52 (0.23)  51.2 (1.93) | 4.0%  4.2%  3.8% |
| Lot to lot  (Three different lots) | 1.20 (0.02)  5.33 (0.15)  48.7 (1.47) | 1.3  2.9  3.0 | 1.28 (0.07)  5.53 (0.06)  50.3 (0.58) | 5.2%  1.0%  1.1% |
| Interference test | There was no substantial interference from ascorbic acid (<50 mg/dl), bilirubin (<50 mg/dl), haemoglobin (<500 mg/dl), cyle (<3000 FTU) and DTT (0.1%), indicating that the assay was resistant to interference from a wide range of biological constituents. | | | |
