# Supplementary material for: A novel assay for improved detection of sputum periostin in patients with asthma
Source: PLoS One. 2023 Feb 10;18(2):e0281356. doi: 10.1371/journal.pone.0281356 (PMC9916630; doi:10.1371/journal.pone.0281356)
Supplement: S2 Table — (DOCX) [file pone.0281356.s003.docx]

**S2 Table. Linearity and recovery testing of assay A and assay B.**

**Recovery test**

|  | Assay A | | | | | | | |
| --- | --- | --- | --- | --- | --- | --- | --- | --- |
|  | Healthy subject | | Asthma patient 1 | | Asthma patient 2 | | Asthma patient 3 | |
| Spiked (ng/mL) | Measured (ng/mL) | Recovery | Measured (ng/mL) | Recovery | Measured (ng/mL) | Recovery | Measured (ng/mL) | Recovery |
| 0.0 | 0.0 |  | 0.0 |  | 0.0 |  | 1.5 |  |
| 1.0 | 1.02 | 102.0% | 1.01 | 101.0% | 0.95 | 95.0% | 2.48 | 98.0% |
| 10.0 | 9.99 | 99.9% | 9.97 | 99.7% | 9.85 | 98.5% | 11.44 | 99.4% |

|  | Assay B | | | | | | | |
| --- | --- | --- | --- | --- | --- | --- | --- | --- |
|  | Healthy subject | | Asthma patient 1 | | Asthma patient 2 | | Asthma patient 3 | |
| Spiked (ng/mL) | Measured (ng/mL) | Recovery | Measured (ng/mL) | Recovery | Measured (ng/mL) | Recovery | Measured (ng/mL) | Recovery |
| 0.0 | 0.0 |  | 0.1 |  | 0.2 |  | 2.1 |  |
| 1.0 | 1.0 | 101.0% | 1.08 | 98.0% | 1.15 | 95.0% | 3.11 | 101.0% |
| 10.0 | 10.10 | 101.0% | 9.99 | 98.9% | 10.18 | 99.8% | 12.15 | 100.5% |

**Linearity test**

|  | Assay A | | |
| --- | --- | --- | --- |
| Sputum sample | Asthma patient 1 | Asthma patient 2 | Asthma patient 3 |
| Dilution ratio | Measured (ng/mL) | Measured (ng/mL) | Measured (ng/mL) |
| 0.01 | 9.7 | 15.8 | 18.0 |
| 0.005 | 4.9 | 7.9 | 8.7 |
| 0.0025 | 2.3 | 4.0 | 4.7 |
| 0.00125 | 1.2 | 1.9 | 2.0 |
| Pearson correlation (r) | 0.9996 | 0.9999 | 0.9995 |

|  | Assay B | | |
| --- | --- | --- | --- |
| Sputum sample | Asthma patient 1 | Asthma patient 2 | Asthma patient 3 |
| Dilution ratio | Measured (ng/mL) | Measured (ng/mL) | Measured (ng/mL) |
| 0.01 | 17.6 | 18.6 | 20.4 |
| 0.005 | 8.6 | 9.3 | 10.1 |
| 0.0025 | 4.8 | 4.7 | 5.3 |
| 0.00125 | 2.6 | 2.3 | 2.7 |
| Pearson correlation (r) | 0.9995 | 1.0000 | 0.9999 |
