# Supplementary material for: A novel assay for improved detection of sputum periostin in patients with asthma
Source: PLoS One. 2023 Feb 10;18(2):e0281356. doi: 10.1371/journal.pone.0281356 (PMC9916630; doi:10.1371/journal.pone.0281356)
Supplement: S3 Table — (DOCX) [file pone.0281356.s004.docx]

**S3 Table. Patient characteristics of cohort 2.**

| **Cohort 1** | **Healthy** |  | **Asthma** |  | ***Mann-Whitney Test (*or Chi^2^)*** |
| --- | --- | --- | --- | --- | --- |
|  | **Median (IQR)** | ***n*** | **Median (IQR)** | ***n*** | ***P*** |
| **Sex (% female)** | 53% | 15 | 50% | 30 | 0.83* |
| **Age (years)** | 36 (31-53) | 15 | 51 (43-62) | 30 | 0.14 |
| **BMI (kg/m^2^)** | 24.2 (20.7-27.8) | 15 | 28.5 (23.0-33.8) | 30 | 0.06 |
| **ICS (µg/day)** | 0 (0-0) | 15 | 825 (500-1000) | 30 | <0.0001 |
| **OCS (% taking)** | 0% | 15 | 37% | 30 | 0.007* |
| **FEV_1_ (% predicted)** | 98.0 (90.0-103.0) | 15 | 68.5 (44.8-80.0) | 22 | <0.0001 |
| **FVC (% predicted)** | 94.0 (89.0-101.0) | 15 | 83.5 (69.0-88.5) | 22 | 0.0002 |
| **FEV_1_/FVC (ratio)** | 0.83 (0.77-0.89) | 15 | 0.68 (0.50-0.76) | 22 | <0.0001 |
| **Bronchial reversibility (%)** | 2 (0-4) | 15 | 12 (7.3-25.5) | 21 | <0.0001 |
| **FeNO (ppb)** | - |  | 29.0 (12.5-45.5) | 16 | - |
| **IgE (KU/l)** | - |  | 83 (20-309) | 26 | - |
| **Atopy (% positive skin prick test)** | 54% | 13 | 76% | 29 | 0.15* |
| **Sputum periostin Assay A (ng/ml)** | 0.0 (0.0-0.2) | 15 | 0.05 (0.0-0.7) | 30 | 0.43 |
| **Sputum periostin Assay B (ng/ml)** | 0.1 (0.0-0.2) | 15 | 0.5 (0.1-1.9) | 30 | 0.005 |
| **Blood eosinophils (****x10^3^ cells/µl)** | - |  | 0.25 (0.02-0.50) | 30 | - |
| **Sputum eosinophils (%)** | 0.3 (0-0.5) | 15 | 3.6 (0.2-11.0) | 30 | 0.009 |
| **Sputum neutrophils (%)** | 26.8 (15.4-51.8) | 15 | 54.8 (30.6-74.9) | 30 | 0.007 |
| **Sputum IL-4 (pg/ml)** | 2.7(2.7-4.9) | 9 | 4.9 (2.7-8.0) | 23 | 0.14 |
| **Sputum IL-13 (pg/ml)** | 0.72 (0.32-1.26) | 11 | 0.98 (0.60-1.81) | 26 | 0.065 |
| **Serum periostin (ng/ml)** | 57 (49-63) | 14 | 58 (50-87) | 12 | 0.75 |
