# Supplementary material for: A novel assay for improved detection of sputum periostin in patients with asthma
Source: PLoS One. 2023 Feb 10;18(2):e0281356. doi: 10.1371/journal.pone.0281356 (PMC9916630; doi:10.1371/journal.pone.0281356)
Supplement: S4 Table — (DOCX) [file pone.0281356.s005.docx]

**S4 Table. Detection levels of Assay A and Assay B at follow-up visits.**

|  | Visit 1 | | Visit 2 (week 4) | | Visit 4 (week 23) | | Visit 6 (week 58) | |
| --- | --- | --- | --- | --- | --- | --- | --- | --- |
| *n* | 80 | | 64 | | 50 | | 39 | |
|  | Assay A | Assay B | Assay A | Assay B | Assay A | Assay B | Assay A | Assay B |
| Detection level | 44% | 90% | 34% | 86% | 36% | 92% | 38% | 82% |
| Periostin ng/ml Median (IQR) | 0 (0-0.4) | 0.4 (0.125-0.7) | 0 (0-0.2) | 0.35 (0.1-1.5) | 0 (0-0.3) | 0.4 (0.2-1.4) | 0 (0-0.6) | 0.3 (0.4-1.6) |
