# Supplementary material for: A novel assay for improved detection of sputum periostin in patients with asthma
Source: PLoS One. 2023 Feb 10;18(2):e0281356. doi: 10.1371/journal.pone.0281356 (PMC9916630; doi:10.1371/journal.pone.0281356)
Supplement: S5 Table — (DOCX) [file pone.0281356.s006.docx]

**S5 Table. Correlations between serum and sputum periostin with sputum cytokines in cohort 2.**

| **Cohort 2** | **Sputum periostin**  **(Assay A)** | | **Sputum periostin**  **(Assay B)** | | **Serum periostin** | |
| --- | --- | --- | --- | --- | --- | --- |
| *Correlation vs* | *Spearman r* | *P* | *Spearman r* | *p* | *Spearman r* | *P* |
| Sputum periostin (Assay A) |  |  | **0.808** | **<0.0001** | 0.175 | 0.586 |
| Sputum periostin (Assay B) | **0.808** | **<0.0001** |  |  | 0.288 | 0.362 |
| Serum periostin | 0.175 | 0.586 | 0.288 | 0.362 |  |  |
| Sputum IL-4 | 0.305 | 0.157 | 0.193 | 0.377 | -0.226 | 0.558 |
| Sputum IL-13 | **0.417** | **0.034** | **0.548** | **0.004** | 0.328 | 0.353 |
