# Supplementary figures and images for: A novel assay for improved detection of sputum periostin in patients with asthma
Source: PLoS One. 2023 Feb 10;18(2):e0281356. doi: 10.1371/journal.pone.0281356 (PMC9916630; doi:10.1371/journal.pone.0281356)

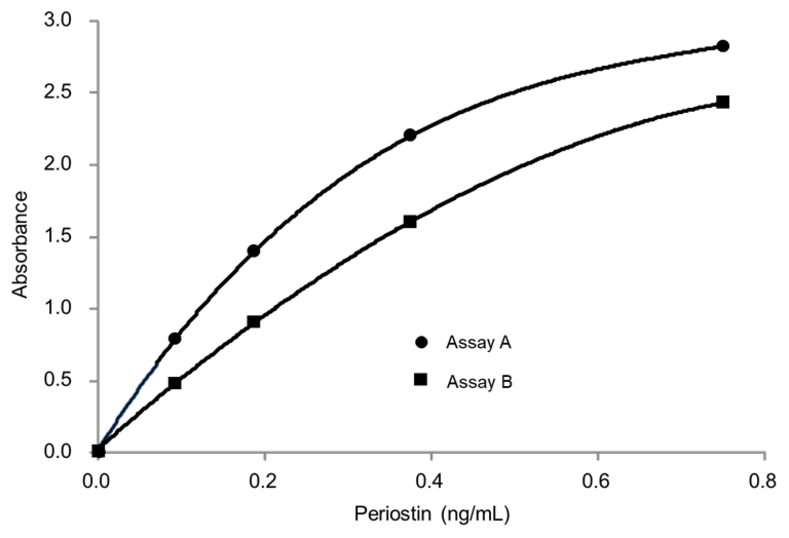

Supplement: S1 Fig — (TIF) [file pone.0281356.s007.tif]

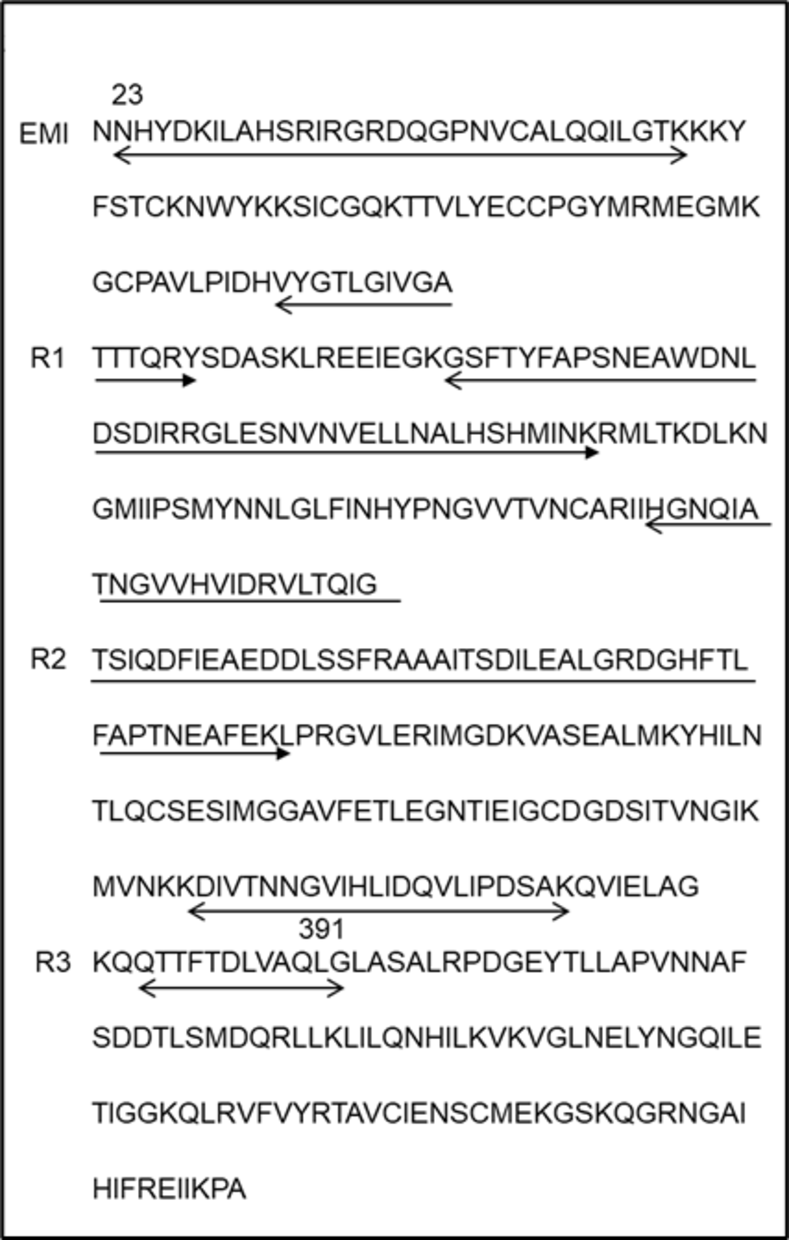

Supplement: S2 Fig — The peptides detected by MALDI/TOF-MS are displayed by arrows. The locations of Asn23 and Lys391 are shown. The 37kDa periostin product was purified from human serum and subjected to MALDI/TOF-MS analysis for further identification. The peptide sequence of the band corresponding to 37 kDa detected by MALDI/TOF-MS analysis covered the amino acid sequence from Asn23 to Leu391 of periostin. The predicted molecular weight of the peptide from Asn23 to Leu391 is 40,141, compatible with the migrated band at 37 kDa. (TIF) [file pone.0281356.s008.tif]

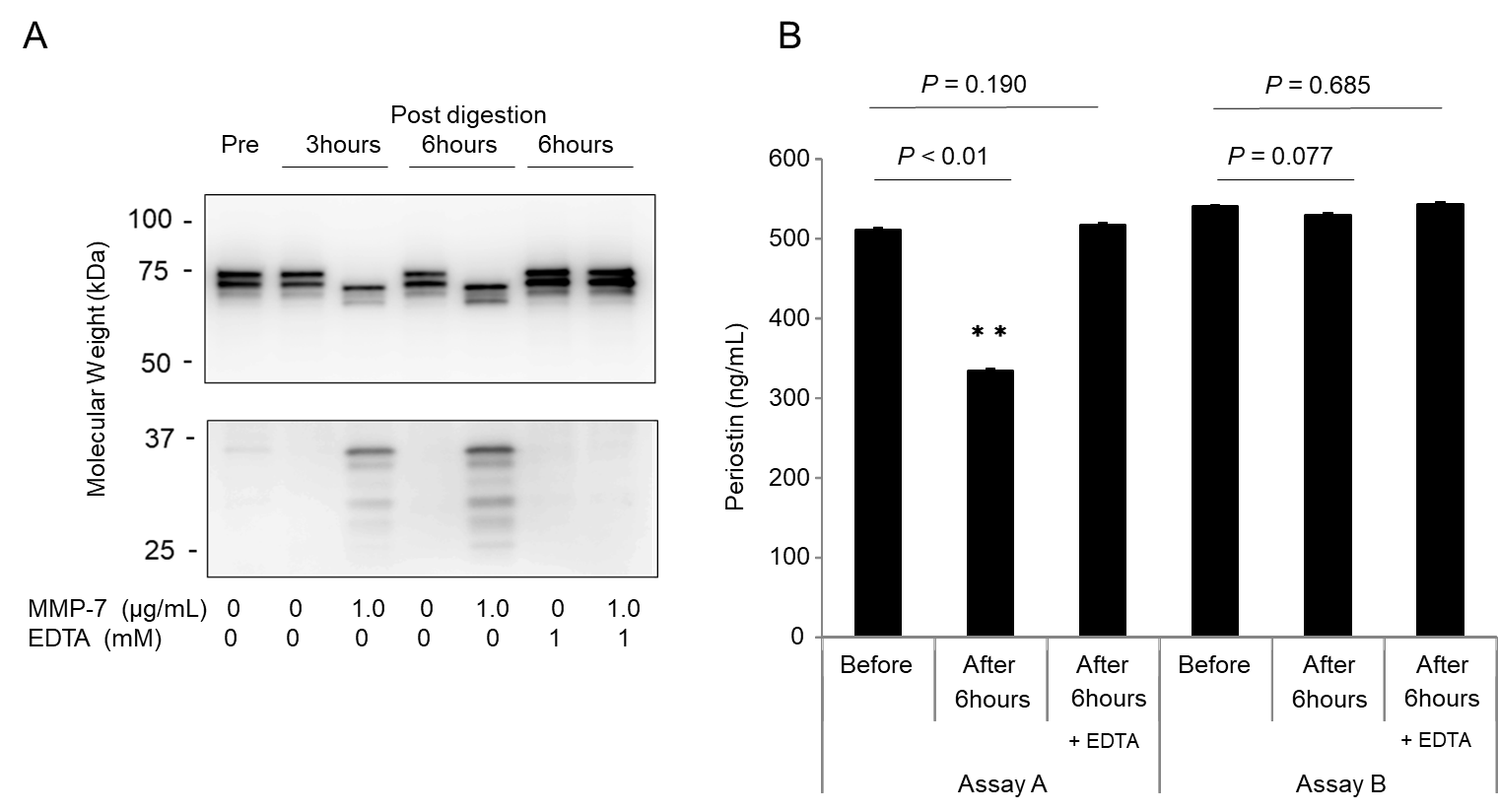

Supplement: S3 Fig — Recombinant periostin was incubated with 1 μg/mL of MMP-7 for 6 hours in the presence or absence of 1 mM EDTA. A Western blot showing periostin staining (A) and periostin values estimated by Assay A and Assay B (B) is depicted. (TIF) [file pone.0281356.s009.tif]

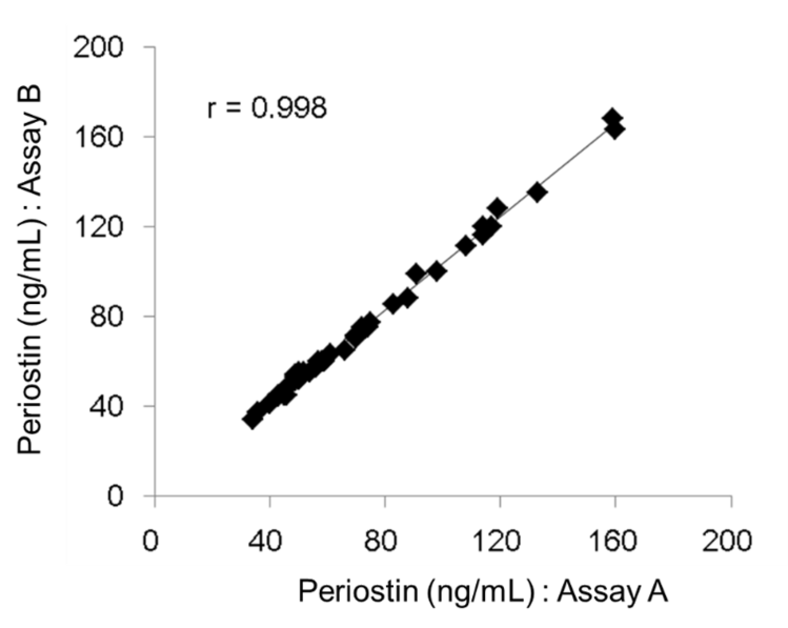

Supplement: S4 Fig — Serum periostin values were estimated in asthma patients using Assay A and Assay B. (TIF) [file pone.0281356.s010.tif]

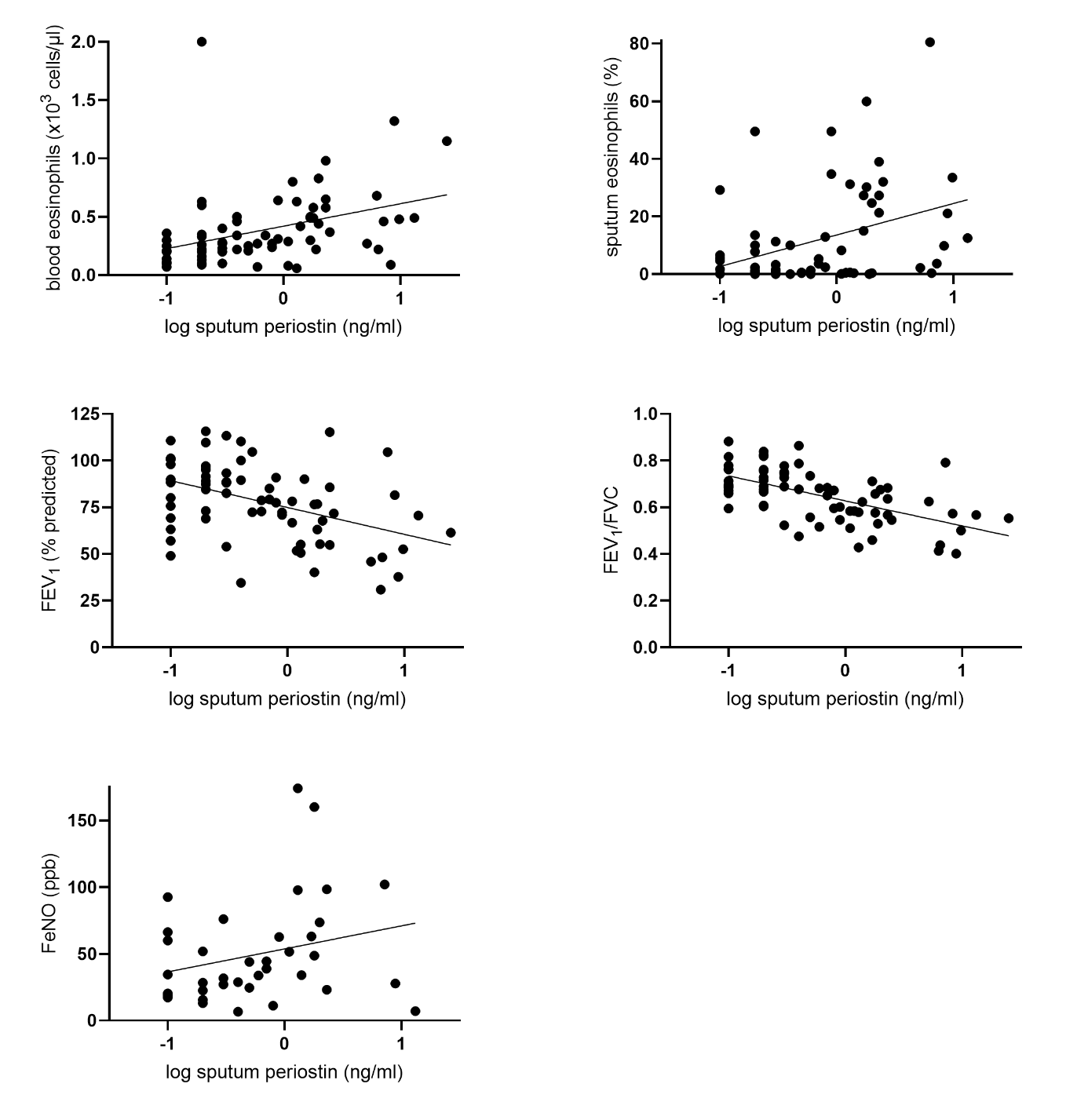

Supplement: S5 Fig — Plots show log sputum periostin levels measured by Assay B in patients with asthma from the BIOAIR study. Undetectable sputum periostin values are therefore not included in this analysis. Each plot is fitted with a linear regression line, the slope of which deviated significantly from zero (p<0.01) for all parameters shown apart from FeNO. (TIF) [file pone.0281356.s011.tif]
