# Supplementary material for: A novel assay for improved detection of sputum periostin in patients with asthma
Source: PLoS One. 2023 Feb 10;18(2):e0281356. doi: 10.1371/journal.pone.0281356 (PMC9916630; doi:10.1371/journal.pone.0281356)
Supplement: S1 Raw images — (PDF) [file pone.0281356.s012.pdf]

S1_raw-images

A novel assay for improved detection of sputum periostin in patients with asthma

Short title: New sputum periostin assay

Junya Ono[1], Masayuki Takai[1], Ayami Kamei[1], Shoichiro Ohta[2], Parameswaran Nair[3], Kenji Izuhara[4], Sven-Erik Dahlén[5], Anna James[5*], on behalf of the BIOAIR consortium^

1. Shino-Test Corporation Ltd., Sagamihara, Japan
2. Department of Laboratory Medicine, Saga Medical School, Saga, Japan
3. Department of Medicine, Division of Respirology, McMaster University and St Joseph's Healthcare, Hamilton, Ontario, Canada
4. Division of Medical Biochemistry, Department of Biomolecular Sciences, Saga Medical School, Saga, Japan
5. Experimental Asthma and Allergy Research, Institute of Environmental Medicine, Karolinska Institutet, Stockholm, Sweden

*Corresponding author

Anna James

Experimental Asthma and Allergy Research

Institute of Environmental Medicine

Karolinska Institutet

171 77 Stockholm

Sweden

Anna.James@ki.se

^Membership of the BIOAIR Consortium is provided in Acknowledgements

Raw images for Figure 1A

Order of loading

1. Molecular weight marker
2. Recombinant periostin
3. Serum periostin
4. Recombinant periostin without immunoprecipitation

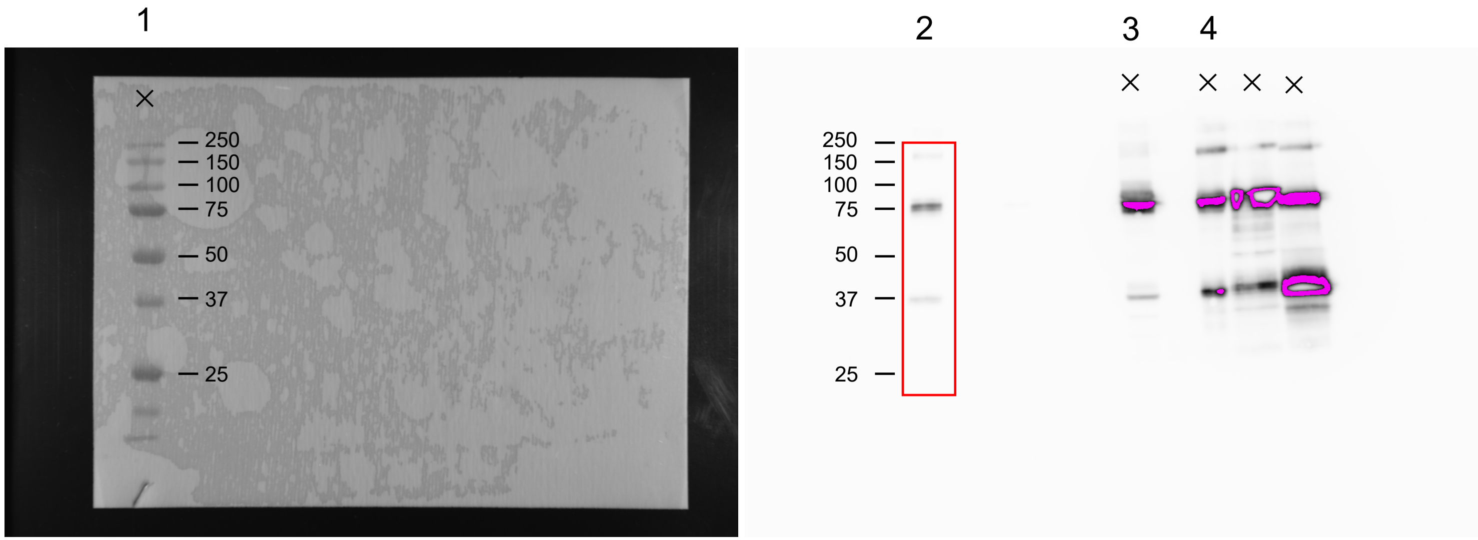

IP:SS19C
Blot:SS19C

Left image: Molecular weight markers was captured on the same membrane.
Right image: The image was captured using ImageQuant LAS camera system (GE Healthcare)
Experimental date: 20, August, 2010

Raw images for Figure 1B

Order of loading
1. Molecular weight marker
2. SS16A
3. SS17B
4. SS18A
5. SS19A
6. SS19C
7. SS19D
8. SS20A
9. SS21A
10. SS21B
11. Polyclonal Abs

(Items 2–11: IP mAb)

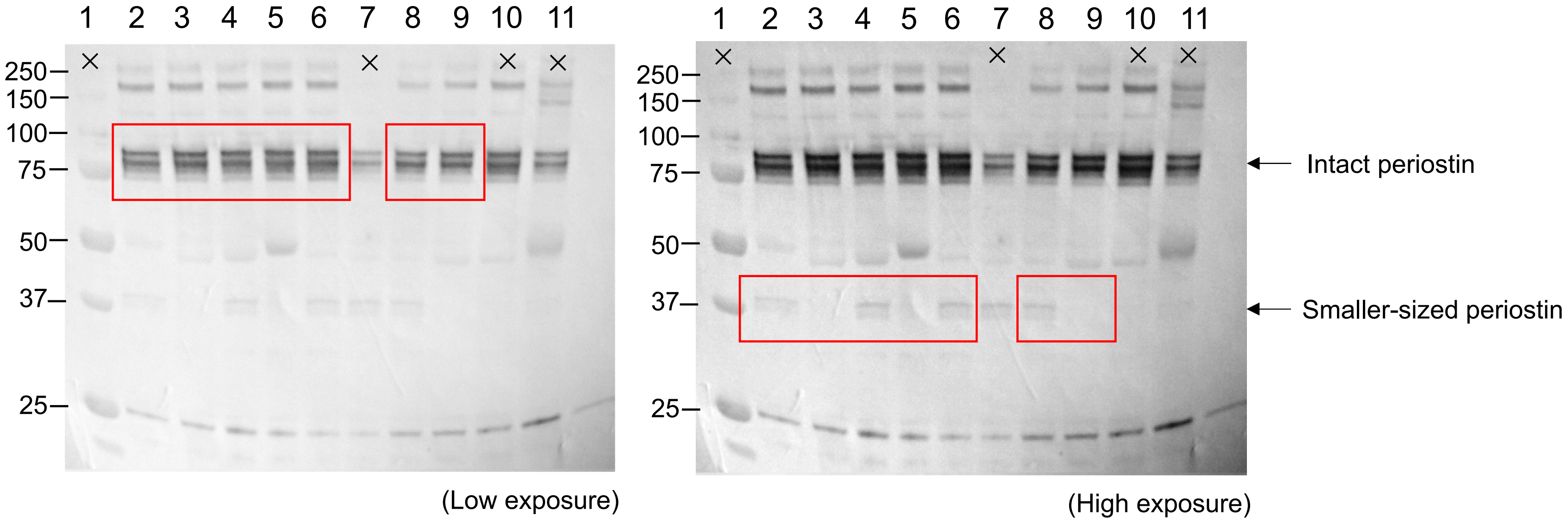

PVDF was stained with 3,3-diaminobenzidine (DAB), and then the image was captured using Fluorchem FC2 image system (Alpha Innotech, USA).
Experimental data: 05, March, 2010

Raw images for Figure 1D

Order of loading

1. Molecular weight marker
2. Recombinant periostin

IP:SS18A
Blot:SS17B

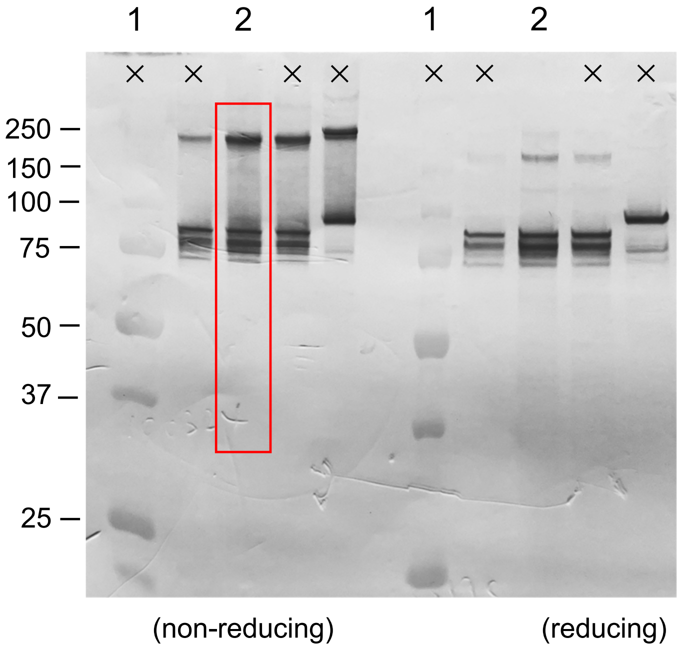

IP:SS18A
Blot:SS19C

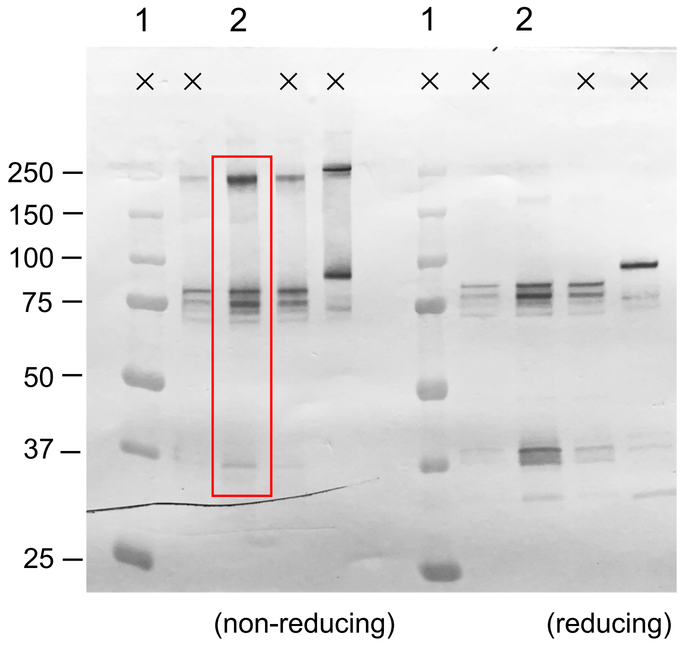

PVDF was stained with 3,3-diaminobenzidine (DAB), and then the image was captured using Fluorchem FC2 image system (Alpha Innotech, USA).
Experimental date: 09, March, 2010 (Left image), 24, March, 2010 (Right image)

Raw images for Figure 5

Order of loading

1. Molecular weight marker
2. Recombinant periostin
3. Sputum sample #1
4. Sputum sample #2
5. Sputum sample #3
6. Sputum sample #4
7. Sputum sample #5
8. Sputum sample #6
9. Sputum sample #7
10. Sputum sample #8
11. Sputum sample #9
12. Sputum sample #10

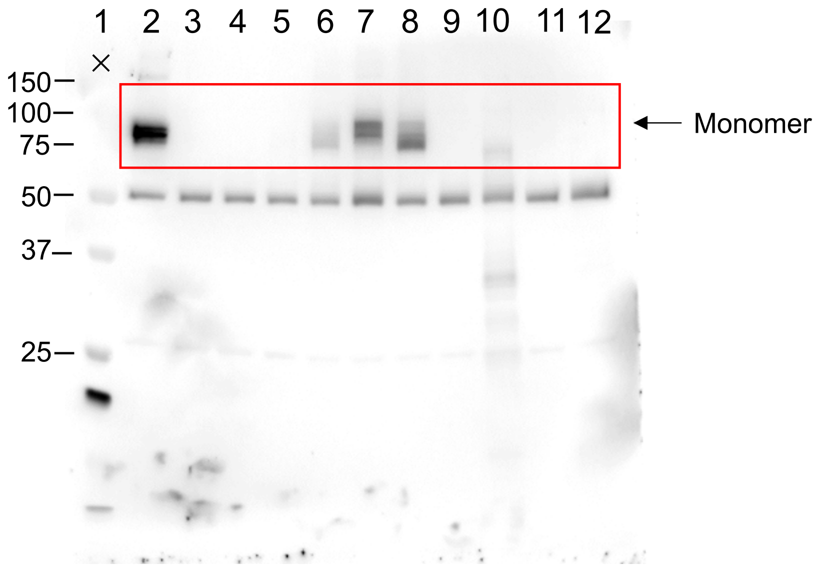

IP:SS18A
Blot:SS17B

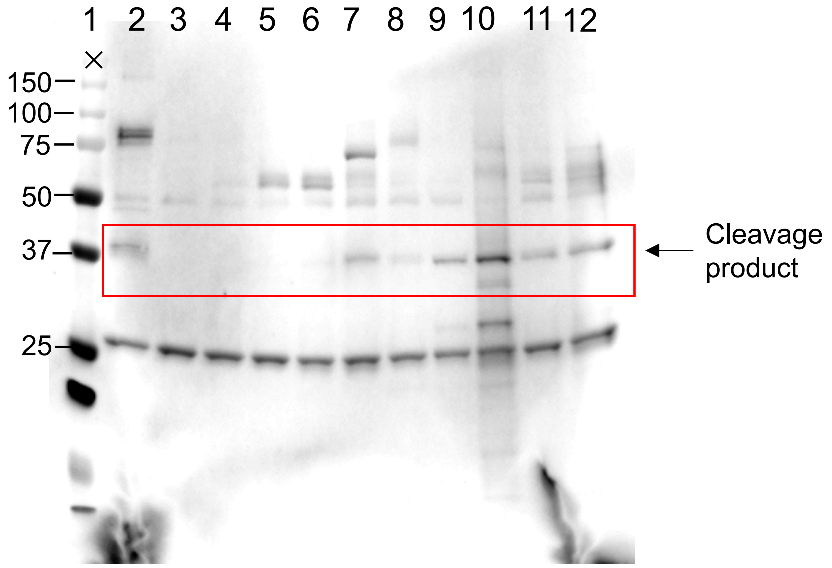

IP:SS18A
Blot:SS19C

The image was captured using Fluorchem FC2 image system (Alpha Innotech, USA).
Experimental date: 08, June. 2017

Raw images for S3 Figure

Order of loading

Lane	Digestion time (hr.)	MMP-7 (μg/mL)	EDTA (mM)
1	Molecular weight marker		
2	0	0	0
3	3	0	0
4	3	1.0	0
5	6	0	0
6	6	1.0	0
7	6	0	1
8	6	1.0	1
9	Molecular weight marker		

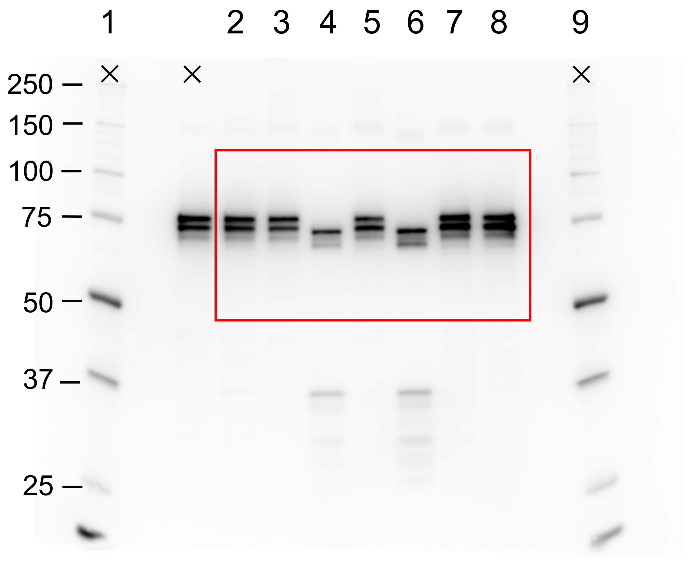

(Low exposure)

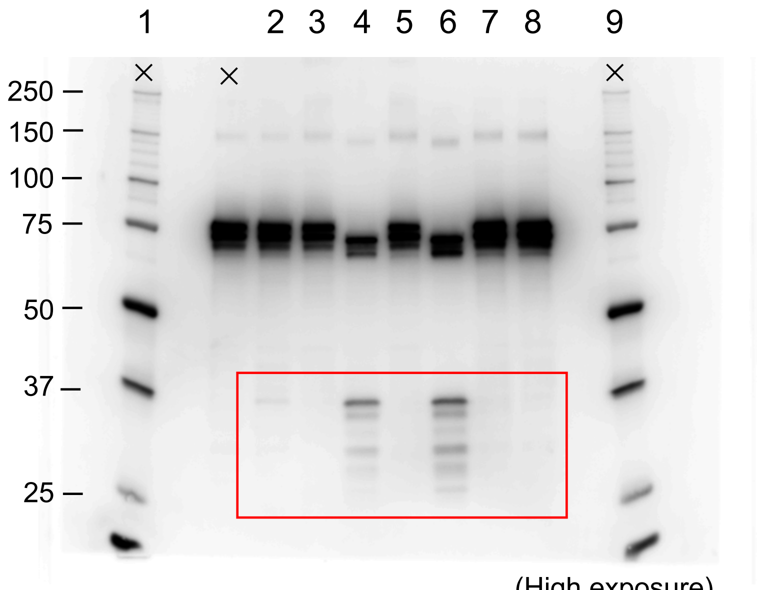

(High exposure)

The image was captured using Fluorchem FC2 image system (Alpha Innotech, USA).
Experimental date: 06, September, 2020
